# The ERα/KDM6B regulatory axis modulates osteogenic differentiation in human mesenchymal stem cells

**DOI:** 10.1038/s41413-021-00171-z

**Published:** 2022-01-07

**Authors:** Zhenqing Liu, Hye-Lim Lee, Jin Sook Suh, Peng Deng, Chang-Ryul Lee, Olga Bezouglaia, Mojan Mirnia, Vivian Chen, Michael Zhou, Zhong-Kai Cui, Reuben H. Kim, Min Lee, Tara Aghaloo, Christine Hong, Cun-Yu Wang

**Affiliations:** 1 Division of Oral Biology and Medicine, School of Dentistry, University of California, Los Angeles (UCLA), Los Angeles, CA 90095 USA; 2 Department of Anatomy & Neurobiology, University of California, Irvine (UCI), Irvine, CA 92697 USA; 3 Department of Orofacial Sciences, School of Dentistry, University of California, San Francisco (UCSF), San Francisco, CA 94143 USA; 4 School of Dentistry, University of California, San Francisco (UCSF), San Francisco, CA 94143 USA; 5 Division of Diagnostic and Surgical Sciences, School of Dentistry, University of California, Los Angeles (UCLA), Los Angeles, CA 90095 USA; 6 School of Dentistry, University of California, Los Angeles (UCLA), Los Angeles, CA 90095 USA; 7 Division of Advanced Prosthodontics, School of Dentistry, University of California, Los Angeles (UCLA), Los Angeles, CA 90095 USA; 8 Division of Constitutive and Regenerative Sciences, School of Dentistry, University of California, Los Angeles, (UCLA), Los Angeles, CA 90095 USA; 9 Department of Bioengineering, University of California, Los Angeles (UCLA), Los Angeles, CA 90095 USA

**Keywords:** Bone, Metabolic disorders

## Abstract

Osteoporosis is a highly prevalent public health burden associated with an increased risk of bone fracture, particularly in aging women. Estrogen, an important medicinal component for the preventative and therapeutic treatment of postmenopausal osteoporosis, induces osteogenesis by activating the estrogen receptor signaling pathway and upregulating the expression of osteogenic genes, such as bone morphogenetic proteins (BMPs). The epigenetic regulation of estrogen-mediated osteogenesis, however, is still unclear. In this report, we found that estrogen significantly induced the expression of lysine-specific demethylase 6B (KDM6B) and that KDM6B depletion by *shRNA*s led to a significant reduction in the osteogenic potential of DMSCs. Mechanistically, upon estrogen stimulation, estrogen receptor-α (ERα) was recruited to the *KDM6B* promoter, directly enhancing *KDM6B* expression. Subsequently, KDM6B was recruited to the *BMP2* and *HOXC6* promoters, resulting in the removal of H3K27me3 marks and activating the transcription of *BMP2* and *HOXC6*, the master genes of osteogenic differentiation. Furthermore, we found that estrogen enhanced DMSC osteogenesis during calvarial bone regeneration and that estrogen’s pro-osteogenic effect was dependent on KDM6B in vivo. Taken together, our results demonstrate the vital role of the ERα/KDM6B regulatory axis in the epigenetic regulation of the estrogen-dependent osteogenic response.

## Introduction

Osteoporosis is a systemic disorder characterized by skeletal microarchitectural deterioration and reduced bone mass leading to fracture risks. The disease is multifactorial and caused by an imbalance between bone formation and resorption mediated by osteoblasts and osteoclasts, respectively.^1^ Estrogen deficiency in postmenopausal women particularly aggravates skeletal fragility; it is estimated that a 50-year-old Caucasian woman has a 50% risk of skeletal fracture.^2^ Antiresorptive agents such as bisphosphonates or denosumab are commonly used as treatment options for postmenopausal women with increased fracture risk.^3^ However, severe adverse effects of antiresorptive agents, including atypical femur fracture or medication-related osteonecrosis of the jaw, have been reported, causing a decline in clinical use.^4–6^ An alternative therapeutic option is hormonal therapy including estrogen, but various side effects limit its long-term use.^7–9^ Therefore, elucidating the precise genetic and epigenetic regulatory mechanisms of estrogen-mediated osteogenic differentiation of progenitor cells is critical to achieving improved clinical outcomes for treating postmenopausal osteoporosis.

Human mesenchymal stromal cells (MSCs) are progenitor cells with the following characteristics: (1) the ability to self-renew and differentiate into various cell types, including osteoblasts, chondrocytes, and adipocytes; (2) ease of isolation; and (3) lack of immunogenicity, making them indispensable tools in tissue engineering and regenerative therapy.^10,11^ Although MSCs isolated from adult bone marrow (BMSCs) are best known, current studies using MSCs derived from dental tissues in bone tissue engineering have demonstrated promise for multiple clinical applications, as dental mesenchymal stromal cells (DMSCs) display similar characteristics to BMSCs.^12,13^ In fact, compared to BMSCs, DMSCs may be more advantageous as DMSCs are more readily available without significant risk to the donor from discarded dental tissues such as third molars or primary teeth. Furthermore, these cells exhibit increased mineralization potential, a faster proliferation rate, and a higher number of stem/progenitor cells than BMSCs.^14,15^

Estrogen (E2), a naturally occurring steroid, is best known for its function in reproductive organ development.^16^ However, as exhibited in the clinical manifestation of postmenopausal osteoporosis, estrogen also plays an important role in bone homeostasis and has been consistently shown to be protective of bone in all sexes.^17^ While estrogen deficiency rapidly leads to systemic osteoporosis in both long bones and craniofacial bones, the addition of exogenous estrogen can enhance the osteogenic potential of MSCs, leading to restoration of bone loss.^18,19^ On a cellular level, estrogen directs MSC differentiation into osteogenic lineages and osteoblast proliferation, leading to an overall increase in bone mineral density.^20–23^ Estrogen induces these pro-osteogenic effects by binding to estrogen receptors, estrogen receptor-α (ERα) and estrogen receptor-β (ERβ), which then act as transcription factors to control the expression of genes related to bone formation.^24^ While estrogen signaling is regulated through the balance between the activities of ERα and ERβ, ERα has been shown to be more important in estrogen-mediated osteogenesis. Zhou et al. reported that estrogen stimulates bone morphogenetic protein-2 (*BMP2*) gene transcription in mouse MSCs, leading to enhanced bone formation. Mechanistically, estrogen-activated ERα and ERβ both bind to estrogen-responsive element (ERE) binding sites in the *BMP2* promoter. However, ERα was substantially more efficacious than ERβ at activating the *BMP2* promoter.^20^ In another study by Chen et al., primary MSCs were isolated from postmenopausal women with osteoporosis. When these cells were treated with estrogen, *ERα* and alkaline phosphatase (*ALP*) mRNA levels were upregulated, while *ERβ* expression remained unchanged, suggesting that estrogen could promote the osteogenic potential of MSCs from osteoporotic patients via ERα activity.^21^ As such, the regulatory mechanisms of estrogen-mediated bone formation at the transcriptional level are understood. However, the key epigenetic players in estrogen-mediated osteogenesis remain unknown.

Stem cell fate determination requires a delicate orchestration of genetic and epigenetic programs. Epigenetic modifiers are largely responsible for the elaborate orchestration of gene activation and inhibition at specific time points, directed toward the terminally differentiated phenotype of MSCs. Among various epigenetic regulators, histone methylation has recently emerged as an integral mechanism in MSC lineage commitment.^25,26^ Specifically, histone methylation status is managed by two opposing enzymes, histone methyltransferases, and histone demethylases.^27^ A high density of repressive epigenetic marks, such as H3K27me3 and H3K9me3, on the promoter region inhibits gene expression.^28^ When these inhibitory epigenetic signatures on lineage-specific genes are erased by histone demethylases, silenced genes become activated to drive stem cell differentiation into specific lineages.^29^

KDM6B, a member of the Jumonji-C domain-containing histone demethylase family, reduces H3K27me3 to H3K27me1 at the pericentric heterochromatin. KDM6B’s diverse functions in stem cell differentiation and development are becoming increasingly elucidated. KDM6B selectively modulates H3K27me3 silencing epigenetic signatures in target genes and lineage-specific genes to direct stem cells to a specific lineage fate.^30^ A decade ago, KDM6B’s role in cell differentiation was first identified in ectodermal differentiation, including epidermal differentiation and neural stem cell differentiation in human cells.^31,32^ Studies have shown that KDM6B, by directly upregulating the expression of neurogenic genes, including *Pax6*, *Nestin*, and *Sox*, is critical for the neural commitment of embryonic stem cell (ESC)-derived neural stem cells.^32^ During neural differentiation, KDM6B was also found to interact with Smad2/3 and Smad1 to activate the transcription of target neuronal genes.^33,34^ Other studies revealed the involvement of KDM6B in the formation of the endoderm by either modulating the WNT signaling pathway or binding to T-box transcription factors to drive ESCs into a definitive endodermal lineage.^35,36^ Our previous studies focused on MSC differentiation characterized KDM6B as a major epigenetic regulator in MSC osteogenic and odontogenic differentiation.^25,37^ Moreover, we recently showed that alcohol-induced inhibition of KDM6B is responsible for dysregulation of DMSC osteogenic differentiation following exposure to alcohol.^38^ Less is known, however, about the role of KDM6B in estrogen-dependent MSC osteogenic commitment. Therefore, this study examined the epigenetic program responsible for the estrogen-dependent osteogenic response by investigating KDM6B and elucidating the mechanism by which KDM6B regulates estrogen-mediated stem cell differentiation.

## Results

### Estrogen induces osteogenic differentiation and KDM6B expression in DMSCs

**Fig. 1:**
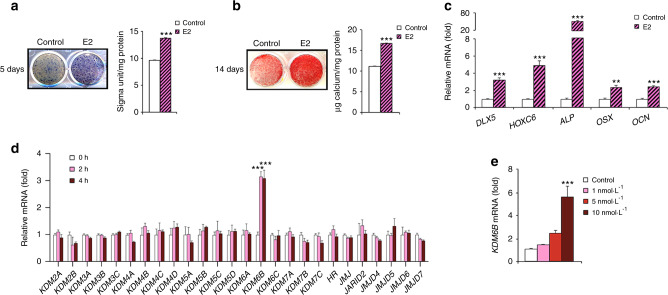
E2 increases osteogenic differentiation and induces KDM6B in DMSCs. **a** Alkaline phosphatase staining and quantification after 5 days of treatment in osteogenic media with and without E2. **b** Alizarin Red S staining and quantification after 14 days of treatment in osteogenic media with and without E2. **c** qRT-PCR of osteogenic genes (*DLX5, HOXC6, ALP, OSX, OCN*) upon E2 treatment in DMSCs. **d** qRT-PCR of epigenetic regulatory genes upon E2 (10 nmol·L^–1^) treatment at 2 and 4 h in DMSCs. **e** qRT-PCR of *KDM6B* in different E2 concentrations (1, 5, 10 nmol·L^–1^) at 2 h. Data are presented as the mean ± SEMs (**a**, **b**, *n* = 9, **c**–**e** *n* = 3). For **a** and **b**, the control and E2-treated cells were compared by the two-tailed t test. For **c** and **d**, data are shown as fold expression of target genes after normalization to the control. For **c**, control and E2-treated cells were compared by two-tailed *t*-tests. For **d**, the groups were compared by two-way ANOVA with a Bonferroni post hoc test. For **e**, the groups with different concentrations of E2 were compared by one-way ANOVA with Tukey’s post hoc test. Asterisks were assigned to *P* values with statistical significance (**P < 0.01; ***P < 0.001)

We isolated DMSCs from dental pulpal tissues and found that they could be differentiated into osteoblasts, adipocytes, and chondrocytes in different induction media, although adipogenic differentiation was relatively weak (Supplementary Fig. S1a). The results demonstrated that DMSCs show multipotential differentiation. Previous studies have shown that 17β-estradiol (E2), an inducer of osteogenesis, has significant potential in bone regeneration and repair.^39–41^ To determine whether E2 could promote the osteogenic differentiation of DMSCs, we first induced the cells to undergo osteogenic differentiation in the presence of E2. E2-enhanced osteogenesis in DMSCs from all donors, as demonstrated by increases in ALP activity after treatment for 5 days (Fig. 1a and Supplementary Fig. S1b), mineralization after treatment for 14 days (Fig. 1b and Supplementary Fig. S1c), and the expression of genes associated with osteogenesis, including *DLX5*, *HOXC6*, *ALP*, *OSX*, and *OCN*, after treatment for 4 days (Fig. 1c).

Previously, we reported that osteogenic differentiation was epigenetically regulated by KDM6B in different human MSCs.^25,37^ To investigate the potential roles of epigenetic modulators in E2-induced DMSC osteogenesis, we profiled the expression of 26 epigenetic regulators in DMSCs following E2 (10 nmol·L^-1^) treatment without osteogenic inducing medium (OIM) for 2 and 4 h. qRT-PCR revealed that *KDM6B* was most strongly upregulated by E2 (Fig. 1d). To examine determine *KDM6B* induction by E2 was dose-dependent, we treated DMSCs with different doses of E2 (1, 5, 10 nmol·L^-1^) without OIM for 2 h. qRT-PCR revealed that *KDM6B* expression was significantly upregulated in a dose-dependent manner (Fig. 1e). *KDM6B* mRNA expression was most upregulated after induction with 10 nmol·L^-1^ E2. Our results suggest that KDM6B has a potential role in estrogen’s regulation of human DMSC fate commitment.

### KDM6B is required for induction of osteogenesis mediated by E2

**Fig. 2:**
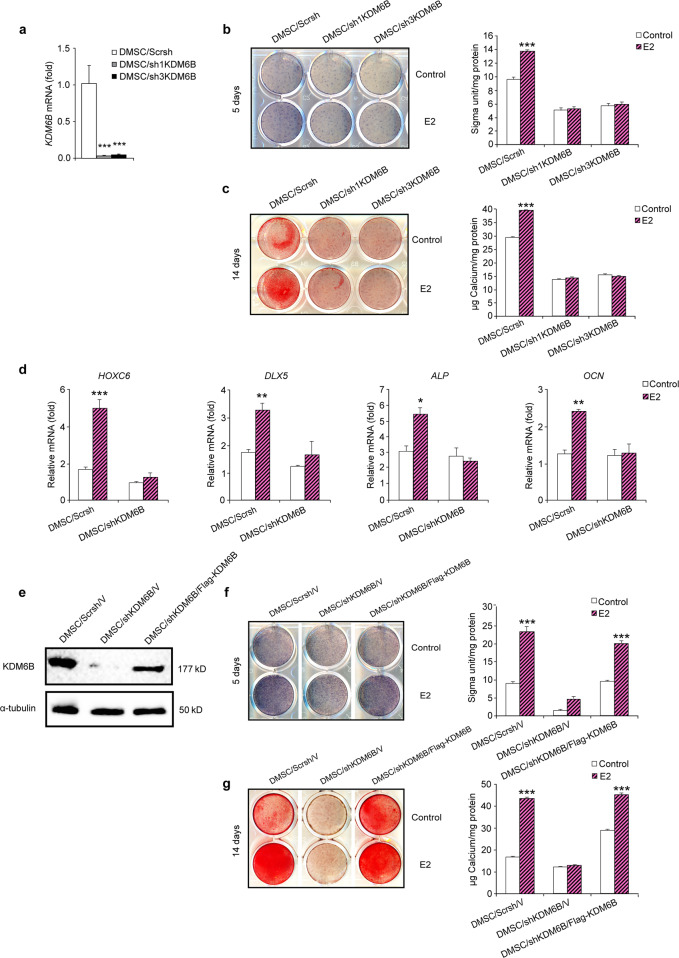
KDM6B is critical for E2-mediated osteogenic differentiation of DMSCs. **a** Depletion of KDM6B in DMSCs via two different shRNAs (*sh1KDM6B* and *sh3KDM6B*) by qRT-PCR. **b** Alkaline phosphatase staining and quantification of KDM6B-depleted DMSCs after 5 days in osteogenic media with and without E2. **c** Alizarin Red S staining and quantification of KDM6B-depleted DMSCs after 14 days in osteogenic media with and without E2. **d** qRT-PCR of osteogenic genes (*HOXC6, DLX5, ALP, OCN*) in the KDM6B-depleted DMSCs following osteogenic media induction with and without E2. **e** Overexpression of KDM6B in KDM6B-depleted DMSCs confirmed by western blot analysis. **f** Alkaline phosphatase staining and quantification of DMSC/*Scrsh/*V, DMSC/*shKDM6B*/V, and DMSC/*shKDM6B*/Flag-*KDM6B*. **g** Alizarin Red S staining and quantification of DMSC/*Scrsh*/V, DMSC/*shKDM6B*/V, and DMSC/*shKDM6B*/Flag-*KDM6B*. Data are presented as the mean ± SEM (*n* = 3). For **a** and **d**, data are shown as fold expression of target genes after normalization to DMSC/*Scrsh*. For **a**, the groups with *shKDM6B* were compared with *Scrsh* by using one-way ANOVA with Tukey’s post hoc test. For **b**, **c**, **d**, **f** and **g**, all the groups were compared by two-way ANOVA with a Bonferroni post hoc test. Asterisks were assigned to *P* values with statistical significance (*P < 0.05; **P < 0.01; ***P < 0.001)

To determine whether KDM6B is required for the E2-mediated osteogenic differentiation of DMSCs, we knocked down KDM6B by transducing lentiviruses expressing *KDM6B* shRNAs. To rule out off-target effects of shRNA, we used two different shRNA sequences (*sh1KDM6B* and *sh3KDM6B*), both of which resulted in depletion of KDM6B in DMSCs, as confirmed by qRT-PCR (Fig. 2a). When the KDM6B-depleted cells, DMSC/*sh1KDM6B* and DMSC/*sh3KDM6B*, were induced to undergo osteogenic differentiation in the presence of E2, these cells failed to both express enhanced ALP (Fig. 2b) and form mineralized nodules after prolonged treatment (Fig. 2c). These cells also exhibited significant suppression of the expression of osteogenic marker genes, including *HOXC6*, *DLX5*, *ALP*, and *OCN*, with the most significant reduction in *HOXC6* gene expression (Fig. 2d), suggesting that KDM6B is required for E2-mediated osteogenic differentiation.

To verify the specific effects of KDM6B on E2-mediated osteogenic differentiation of DMSCs, we restored KDM6B expression in KDM6B knockdown DMSCs. We ectopically introduced Flag-tagged *KDM6B* in DMSCs that harbored *KDM6B* shRNA targeting the 3’ UTR of *KDM6B* mRNA. This shRNA targeted only endogenous KDM6B and not ectopically introduced KDM6B. As expected in the control DMSCs (DMSC/*Scrsh*/V), E2 treatment resulted in significant induction of KDM6B expression, which was strongly suppressed upon the introduction of *KDM6B* shRNA. Flag-tagged *KDM6B* in the DMSC/*shKDM6B*/Flag-*KDM6B* cells restored KDM6B expression to a level similar to that in the DMSC/*Scrsh*/V cells, as confirmed by western blot analysis (Fig. 2e). When the DMSC/*shKDM6B*/Flag-*KDM6B* cells were induced to undergo osteogenic differentiation, the inhibited osteogenic potential of the E2-treated DMSCs due to KDM6B depletion was restored, as demonstrated by ALP activity and Alizarin Red S staining (Fig. 2f, g).

### KDM6B epigenetically regulates E2-mediated *BMP2* and *HOXC6* gene expression in DMSCs by demethylating H3K27me3 marks

**Fig. 3:**
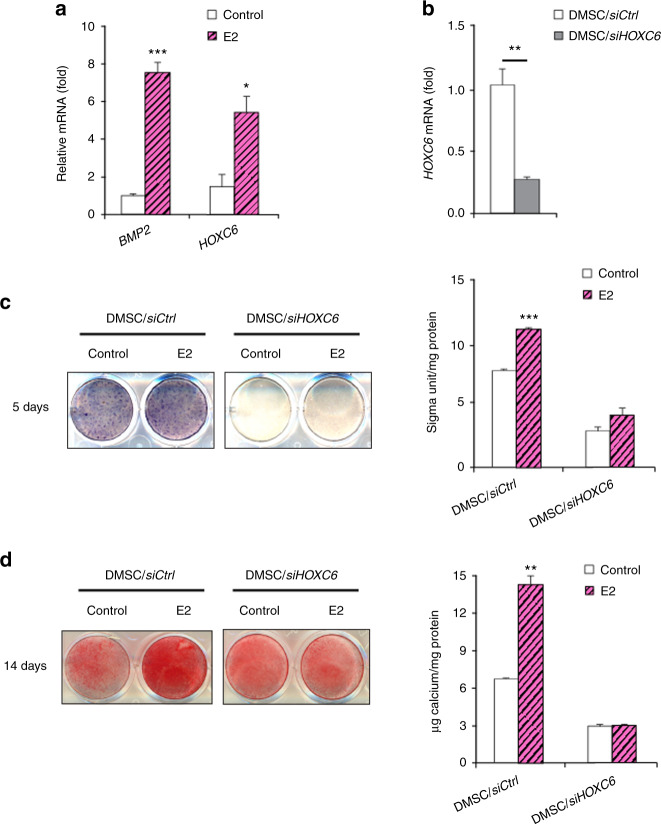
E2-induced BMP and HOXC6 expression in DMSCs. **a** qRT-PCR of *BMP2* and *HOXC6* at 4 h upon E2 treatment in DMSCs. **b** Depletion of HOXC6 in DMSCs via *siRNA* by qRT-PCR. **c** Alkaline phosphatase staining and quantification of HOXC6-depleted DMSCs after 5 days of treatment in osteogenic media with and without E2. **d** Alizarin Red S staining and quantification of HOXC6-depleted DMSCs after 14 days of treatment in osteogenic media with and without E2. Data are presented as the mean ± SEM (*n* = 3). For **a**, data are shown as fold expression of target genes after normalization to the control. For **b**, data are shown as fold expression of target genes after normalization to DMSC/*siCtrl*. For **a** and **b**, two groups were compared by two-tailed *t*-tests. For **c** and **d**, all groups were compared by two-way ANOVA with a Bonferroni post hoc test. Asterisks were assigned to *P* values with statistical significance (*P < 0.05; **P < 0.01; ***P < 0.001)

The *BMP2* and *HOXC6* genes are known to be critical players in osteogenic differentiation.^25,42,43^ Specifically, KDM6B knockdown was shown to inhibit *BMP2* and *HOXC6* expression in DMSCs through epigenetic modifications of histone methylation at the promoter regions of *BMP2* and *HOXC6*, suggesting that KDM6B is vital for osteogenic differentiation.^25^ We explored the effects of estrogen on *BMP2* and *HOXC6* expression and found that E2 treatment significantly upregulated *BMP2* and *HOXC6* expression levels (Fig. 3a). Furthermore, we used *siHOXC6* to knock down HOXC6 expression in DMSCs. The knockdown of *HOXC6* expression was confirmed using qRT-PCR (Fig. 3b). The depletion of HOXC6 in DMSCs resulted in significantly suppressed E2-enhanced osteogenic potential of DMSCs, as demonstrated by ALP activity and mineralization (Fig. 3c, d).

**Fig. 4:**
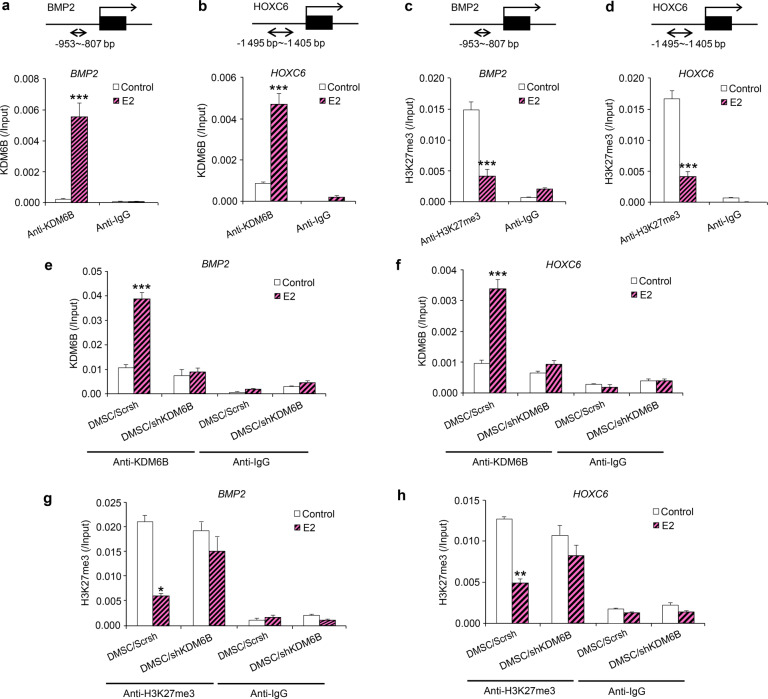
E2 increased BMP2 and HOXC6 expression in DMSCs through the recruitment of KDM6B to promoters and removal of H3K27me3 marks. Occupancy of KDM6B at the *BMP2* (**a**) and *HOXC6* (**b**) promoters, as determined by ChIP, upon E2 treatment. Occupancy of H3K27me3 at the *BMP2* (**c**) and *HOXC6* (**d**) promoters, as determined by ChIP, upon E2 treatment. Occupancy of KDM6B at the *BMP2* (**e**) and *HOXC6* (**f**) promoters in the control and KDM6B-depleted DMSCs, as determined by ChIP, upon E2 treatment. Occupancy of H3K27me3 at the *BMP2* (**g**) and *HOXC6* (**h**) promoters in the control and KDM6B-depleted DMSCs, as determined by ChIP, upon E2 treatment. Data are presented as the mean ± SEM (*n* = 3). All the groups were compared by two-way ANOVA with Bonferroni post hoc test. Asterisks were assigned to *P* values with statistical significance (*P < 0.05; **P < 0.01; ***P < 0.001)

To determine whether E2 induces *BMP2* and *HOXC6* expression through the recruitment of KDM6B to the *BMP2* and *HOXC6* promoters and epigenetic modulation at their promoters, we performed a ChIP assay to assess the physical occupancy of KDM6B and changes in histone methylation status at the *BMP2* and *HOXC6* promoter regions following E2 treatment. We confirmed that E2 treatment led to increased binding of KDM6B to the promoter regions of *BMP2* and *HOXC6* (Fig. 4a, b). Next, we examined whether KDM6B regulates E2-mediated DMSC differentiation through epigenetic modification by assessing the level of H3K27me3 at the *BMP2* and *HOXC6* promoters following E2 treatment. Indeed, upon E2 treatment, demethylation of the silencing epigenetic mark H3K27me3 occurred at the *BMP2* and *HOXC6* promoters, allowing these osteogenic marker genes to be activated (Fig. 4c, d). In contrast, KDM6B occupancy on the promoters of *BMP2* and *HOXC6* was reduced in the KDM6B-depleted DMSCs (Fig. 4e, f), and decreased binding of KDM6B at the promoter regions was associated with increased occurrence of its substrate, H3K27me3 (Fig. 4g, h). Taken together, our data suggest that KDM6B is recruited to the *BMP2* and *HOXC6* promoters and that KDM6B epigenetically regulates *BMP2* and *HOXC6* transcription through the removal of the repressive H3K27me3 mark from the *BMP2* and *HOXC6* promoters following E2 treatment.

### E2 upregulates *KDM6B* gene expression in DMSCs by recruiting ERα to the *KDM6B* promoter

**Fig. 5:**
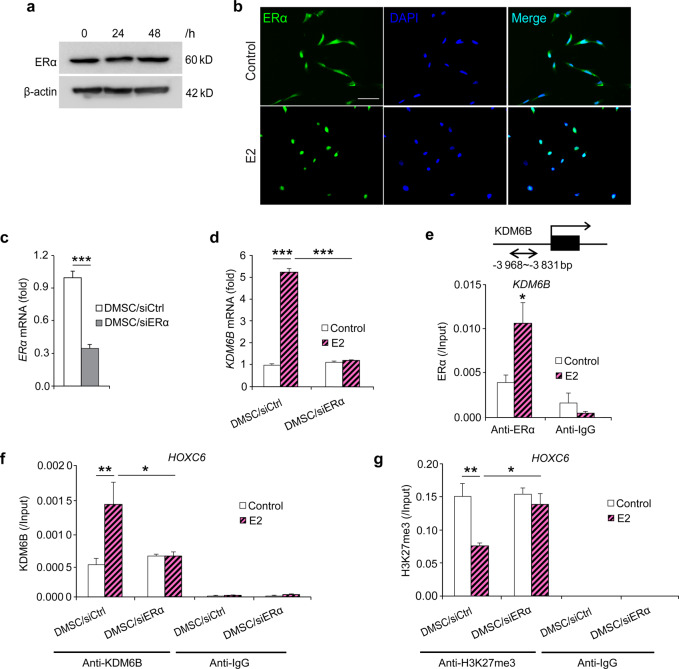
E2-induced KDM6B expression through ERα in DMSCs. **a** ERα protein expression in DMSCs upon E2 treatment at 24 and 48 h by western blots. **b** ERα expression location in DMSCs upon E2 treatment at 4 h by immunofluorescence staining. **c** Depletion of ERα in DMSCs via *siRNA* by qRT-PCR. **d** qRT-PCR of *KDM6B* expression in the control and ERα-depleted DMSCs upon E2 treatment. **e** Occupancy of ERα at the *KDM6B* promoter, as determined by ChIP, upon E2 treatment. **f** Occupancy of KDM6B at the *HOXC6* promoter in the control and ERα-depleted DMSCs, as determined by ChIP, upon E2 treatment. **g** Occupancy of H3K27me3 at the *HOXC6* promoter in the control and ERα-depleted DMSCs, as determined by ChIP, upon E2 treatment. Data are presented as the mean ± SEM (*n* = 3). For **c** and **d**, data are shown as fold expression of target genes after normalization to DMSC/*siCtrl*. For **c**, two groups were compared by two-tailed *t*-tests. For **d**–**g**, all the groups were compared by two-way ANOVA with a Bonferroni post hoc test. Asterisks were assigned to *P* values with statistical significance (*P < 0.05; **P < 0.01; ***P < 0.001)

To further elucidate E2-mediated DMSC osteogenic differentiation, we first investigated whether ERα was involved in E2-mediated DMSC osteogenic differentiation. We found that ERα protein levels were not changed after 24 and 48 h of E2 treatment, as demonstrated by western blots (Fig. 5a). Instead, the nuclear translocation of ERα upon E2 treatment at 4 h was observed (Fig. 5b). Next, we examined whether E2-mediated induction of *KDM6B* is ER dependent. First, we knocked down the expression of ERα using siRNA and assessed upregulation of *KDM6B* expression following E2 treatment. The knockdown efficiency was confirmed by qRT-PCR (Fig. 5c). ERα depletion greatly attenuated the induction of *KDM6B* by E2 (Fig. 5d), indicating that KDM6B expression by E2 is ERα-dependent.

Next, using ChIP assays, we examined whether E2 induces KDM6B expression by recruiting ERα to the promoter region of *KDM6B*. Following E2 treatment, there was an evident increase in the physical occupancy of ERα at the *KDM6B* promoter region (Fig. 5e). Surprisingly, when ERα was depleted using siRNA, this enhanced KDM6B binding to the *HOXC6* promoter region was significantly suppressed (Fig. 5f). This reduced binding of KDM6B was associated with the increased level of its substrate H3K27me3 at the *HOXC6* promoter region in the DMSC/*siERα* cells compared to that of the DMSC/*siCtrl* cells upon E2 treatment (Fig. 5g). Taken together, our data suggest that E2 binds to ERα, which in turn directly regulates *KDM6B* activation and controls KDM6B’s regulation of HOXC6 during E2-mediated osteogenesis.

### KDM6B is required for estrogen-mediated DMSC bone formation in vivo

Our group previously reported the pro-osteogenic effects of estrogen delivery in an animal model of palatal fracture.^44^ To assess the ability of estrogen to induce cell-mediated bone regeneration in a critical-sized defect, we generated calvarial defects with a 3 mm diameter in 8-week-old SCID mice, and Ap-PLGA scaffolds loaded with varying concentrations of E2 (1, 5, 25 μmol·L^-1^) were implanted following DMSC seeding. Micro-CT images demonstrated maximum bone formation at 25 μmol·L^-1^ E2 compared to the scaffold without E2 loading (Supplementary Fig. S2). Quantitative analysis of micro-CT images was performed by calculating bone volume/tissue volume (BV/TV). The results revealed that defects with control scaffolds (0 μmol·L^-1^ E2) showed minimal bone healing of just 3.19% BV/TV only after 8 weeks. In contrast, defects treated with estrogen showed significant increases in defect repair with greater estrogen dosage, amounting to 3.12% at 1 μmol·L^-1^, 5.48% at 5 μmol·L^-1^, and 10.36% at 25 μmol·L^-1^.

**Fig. 6:**
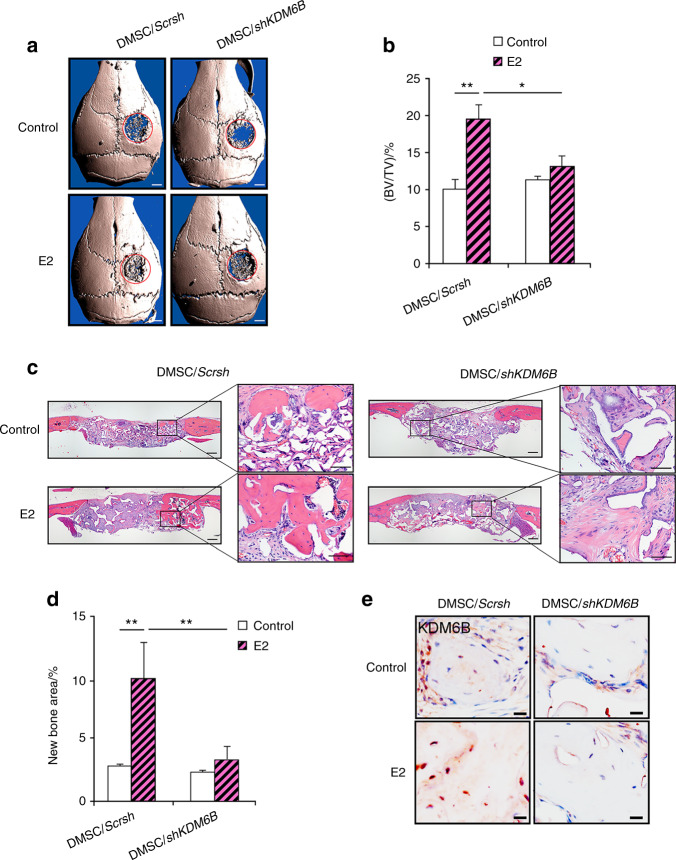
KDM6B is required for E2-mediated DMSC bone formation in vivo. Micro-CT images (**a**) and bone volume quantification (BV/TV) (**b**) of calvarial bone defects with scaffolds containing DMSC/*shKDM6B* and DMSC/*Scrsh* with and without E2. Scale bars, 1 mm. H&E staining images (**c**) and histomorphometric analysis for new bone area within calvarial bone defects (**d**) from mice with transplanted scaffolds seeded with DMSC/*shKDM6B* and DMSC/*Scrsh* with and without E2. Scale bars, 200 μm. **e** Immunostaining of KDM6B in bone tissue formed on scaffolds. Scale bars, 25 μm. Data are presented as the mean ± SEM (*n* = 4). For **b** and **d**, all the groups were compared by two-way ANOVA with a Bonferroni post hoc test. Asterisks were assigned to *P* values with statistical significance (*P < 0.05; **P < 0.01)

Since our in vitro findings suggested that KDM6B plays a critical role in E2-mediated DMSC osteogenesis, we further evaluated whether KDM6B is required for in vivo bone repair using a calvarial defect mouse model. DMSC/*shKDM6B* and DMSC/*Scrsh* were seeded in scaffolds with and without 25 μmol·L^-1^ E2 and subsequently transplanted into 3 mm calvarial critical-sized defects. After 8 weeks, crania with transplanted scaffolds were harvested and prepared for micro-CT and histological analysis. Visualization and quantitative analysis of micro-CT images revealed that E2 treatment was unable to promote further bone repair in the absence of KDM6B compared to that in the DMSC/*Scrsh* cells (Fig. 6a, b). Consistently, H&E staining showed that the loss of KDM6B abrogated the pro-osteogenic effects of E2 in vivo (Fig. 6c, d). Finally, immunostaining confirmed KDM6B induction upon E2 treatment during calvarial defect regeneration (Fig. 6e). Therefore, these results show that E2-mediated bone repair is KDM6B dependent.

## Discussion

Estrogen levels decline with aging in both men and women, and a gradual recession often results in osteoporosis and skeletal fractures. More than 200 million people within the aging population are affected worldwide by osteoporosis. Indeed, estrogen deficiency after menopause causes one in two women to suffer from the condition.^45^ While remarkable progress has been made in the treatment of osteoporosis, elucidation of the precise mechanisms by which estrogen regulates bone formation and maintenance may lead to further improvements in the prevention and treatment of postmenopausal osteoporosis. Estrogen orchestrates a series of molecular mechanisms, particularly the regulation of skeletal growth and bone homeostasis.^46^ Previous studies have established that one such mechanism hinges on estrogen’s ability to increase MSC osteogenic potential.^20,21^ However, the epigenetic events responsible for this process are not understood. Therefore, we explored the role of histone methylation in estrogen-induced MSC osteogenic differentiation and calvarial regeneration. In this study, we demonstrated the vital role of KDM6B in the activation of key osteogenic genes, *BMP2* and *HOXC6*, through dynamic epigenetic remodeling in estrogen-mediated MSC lineage specification.

Our results confirmed the findings from our previous studies using both BMSCs and DMSCs that osteogenic commitment was mediated by KDM6B through *BMP2*, a potent inducer of osteogenic differentiation in MSCs.^25,37^ However, compared to previous studies, our current study using estrogen revealed several key differences in molecular mechanisms. While Agger et al. discovered that KDM6B regulates the expression of *HOX* genes through H3K27me3 demethylation during ESC differentiation,^47^ our previous study by Xu et al. showed that *HOX* family genes were not activated by KDM6B in DMSCs.^37^ The present study, which also used DMSCs, demonstrated that when estrogen is introduced, KDM6B is recruited to the *HOXC6* promoter to activate *HOXC6* transcription, suggesting the complexity of the mechanistic control of KDM6B activation. Moreover, *HOXC6* depletion using shRNA abrogated the estrogen-induced osteogenic potential, indicating that HOXC6 is required to enhance bone formation by estrogen. Another study by Yang et al. reported that KDM6B regulates osteogenesis by modulating H3K27me3 levels in *RUNX2*, the master osteogenic gene, in human MSCs and osteoblasts.^48^ However, we did not observe binding of KDM6B to the *RUNX2* promoter or KDM6B’s regulation of *RUNX2* expression during estrogen-mediated osteogenesis (data not shown), which is consistent with a previous report that there is no KDM6B binding site on the *RUNX2* promoter in human MSCs.^25^ During estrogen-induced osteogenesis, HOX may have a direct role in osteogenesis in a RUNX2-independent manner, as reported by Hassan et al.^49^ Another important difference in this study compared to our previous work is KDM6B’s effect on *DLX5* gene expression. In previous studies, KDM6B depletion did not alter the expression of the *DLX5* gene, a transcription factor that plays critical roles in osteoblast differentiation and craniofacial development.^25,37^ Instead, we showed that BMP-induced KDM4B, a histone demethylase for the repressive H3K9me3 signature, epigenetically regulates and activates *DLX5* expression, leading to MSC osteogenic commitment.^25^ In this study, we found that estrogen-induced *DLX5* expression is significantly suppressed in KDM6B-depleted cells during estrogen-mediated osteogenesis, thus suggesting that regulatory mechanistic variations exist among different pathways.

Previous studies reported that KDM6B can regulate target genes in a demethylase-independent manner.^50,51^ However, our study showed that KDM6B demethylase activity is responsible for the activation of the *BMP2* and *HOXC6* genes, leading to estrogen-induced MSC osteogenic differentiation. As bone homeostasis by estrogen relies on a delicate balance between osteogenesis and adipogenesis, MSC commitment to an osteogenic fate requires the concurrent inhibition of differentiation toward an adipogenic lineage. Our previous study reported that the depletion of KDM6B significantly induced the expression of the master adipogenic transcription factor PPARG, leading to enhanced adipogenic differentiation of human BMSCs.^25^ Similarly, another study showed that KDM6B depletion promoted the differentiation of mouse MSCs into adipocytes, while overexpression of KDM6B inhibited adipogenesis.^52^ These results support the conclusion that KDM6B can direct MSCs into an osteogenic fate by both promoting MSC osteogenic differentiation and restricting adipogenic differentiation. KDM6B’s ability to manage bone-fat balance through estrogen-mediated pathways highlights its potential as a therapy for postmenopausal patients. Beyond its antiadipogenic function, KDM6B likely has additional targets that contribute to estrogen-induced bone formation. For instance, Lu et al. reported that KDM6B removed H3K27me3 at insulin-like growth factor binding protein 5 promoters to activate the target gene, resulting in an MSC-mediated anti-inflammatory effects and bone tissue regeneration.^53^ In addition, KDM6B was found to suppress osteoblast apoptosis by erasing H3K27me3 marks from the antiapoptotic *Bcl-2* gene and regulating Bim phosphorylation.^54^ KDM6B therefore increases osteoblast number and enhances bone formation by both promoting osteoblastic differentiation and stimulating osteoblastic apoptosis. As bone remodeling involves a complex interplay among the immune system, osteoblasts, and osteoclasts, estrogen deficiency has been shown to be linked with increased inflammation, enhanced osteoclast differentiation and decreased osteoblast activity.^1^ As such, the examination of KDM6B’s interaction with inflammatory processes such as NF-κB signaling and the osteoblast apoptotic pathway may reveal KDM6B’s role as a main epigenetic player in estrogen-mediated bone homeostasis and its potential to guard against postmenopausal osteoporosis.

While very little is known about the epigenetic programming of estrogen/ER signaling in MSC osteogenic differentiation, several studies have revealed an association between histone demethylases and ER signaling in breast cancer cells. ERα is a ligand-dependent transcription factor that, upon initiation by estrogen binding, undergoes translocation to the nucleus to modulate the gene transcription machinery.^55^ In the absence of estrogen, specific inhibitory histone methyltransferases place methyl groups on histones to repress constitutive ERα activity.^56^ Upon estrogen stimulation, the histone demethylase KDM1A was found to dismiss these repressive epigenetic marks to induce ER activation, ensuring a ligand-dependent and regulated transcriptional response.^56^ Other studies identified the histone demethylases KDM4B and KDM3A as critical regulators of the ER signaling cascade in cancer cells.^57,58^ Mechanistically, KDM4B directly interacts with the ER and components of the ER signaling system, erasing the inhibitory epigenetic mark H3K9me3 in ERα target sites for the expression of *MYB*, *MYC*, and *CCND1*.^58,59^ In addition, KDM4B’s association with ERα and the transcriptional regulator mixed-lineage leukemia 2 complex was shown to be a critical mechanism for estrogen-induced G1/S transition of the cell cycle in breast cancer.^60^ In breast cancer cells, Svotelis et al. uncovered the participation of KDM6B in estrogen/ERα signaling. Upon E2 induction, KDM6B and ERα work in concert to demethylate the H3K27me3 mark from the poised enhancer region of the ERα-regulated antiapoptotic gene *BCL2*, leading to the prevention of breast cancer cell death.^61^ These findings highlight the dynamic and important role of histone demethylases in the epigenetic control of estrogen and ERα pathways.

Information on the direct targets of ER signaling during bone formation remains limited. Our study revealed that estrogen induces KDM6B expression through the recruitment of ERα to the promoter region of *KDM6B*, indicating the direct regulation of *KDM6B* by ERα at the transcriptional level. This finding underscores the role of ERα signaling in regulating epigenetic modifiers and possibly the overall chromatin landscape, which may constitute a feedforward loop in the cellular function of ERα. Moreover, as shown by KDM4B’s epigenetic modulation of ERα and KDM6B’s physical interaction with ERα in breast cancer cells, there is a possibility that a positive feedback loop exists whereby KDM6B epigenetically promotes ERα transcription.^58,59,61^ Notably, further mechanisms responsible for regulating KDM6B expression during osteogenic differentiation are being elucidated. Emerging evidence has shown that KDM6B expression can be post-transcriptionally regulated by microRNAs. Tang et al. reported that elevated levels of miR-99a in osteoporotic bone marrow suppress osteoblastic commitment and differentiation of MSCs through its inhibitory effects on KDM6B.^62^ Conversely, the inhibition of miR-99a promoted bone formation in vivo. Another study revealed that miR-146a directly associates with the 3′ UTR of *KDM6B* and negatively regulates *KDM6B* function during MSC osteogenic differentiation.^63^ As with miR-99a, inhibition of miR-146a restored KDM6B expression and significantly increased osteogenic potential. Therefore, the identification of KDM6B regulatory mechanisms, such as miR-99a and miR-146a, during estrogen-induced osteogenesis may lead to targeted therapeutic approaches for postmenopausal osteoporosis.

Collectively, our present study demonstrates for the first time that when directly induced by ERα upon estrogen stimulation, the histone demethylase KDM6B epigenetically activates *BMP2* and *HOXC6* transcription during estrogen-mediated osteogenesis. These findings provide a vital epigenetic link between estrogen/ER signaling and bone homeostasis, thereby providing a foundation for future studies.

## Materials and methods

### Cell isolation and culture

The primary DMSCs used in this study were isolated from craniofacial tissues (IRB#13-000241) from four different donors, two males and two females aged 17–24 years old. Cells were grown in phenol red-free alpha-modified Eagle’s medium (αMEM) supplemented with 10% charcoal-stripped fetal bovine serum (FBS) (Omega Scientific, catalog no. FB-04), 100 U·mL^-1^ penicillin and 100 U·mL^-1^ streptomycin in a humidified 5% CO_2_ incubator at 37 °C. The OIM consisted of αMEM supplemented with 5% charcoal-stripped FBS (Omega Scientific, catalog no. FB-04), 100 μmol·L^-1^ ascorbic acid (Sigma-Aldrich, catalog no. A4403), 10 nmol·L^-1^ dexamethasone (Sigma-Aldrich, catalog no. D4902) and 2 mmol·L^-1^ β-glycerophosphate (Sigma-Aldrich, catalog no. G9422) with or without 17 β-estradiol (E2) (Sigma-Aldrich, catalog no. E2758). The medium was changed every 2 days.

### Virus preparation and infection

Viral packaging was performed as previously described.^64^ For viral infection, DMSCs were plated overnight and then infected with retroviruses or lentiviruses together with polybrene (6 μg·mL^-1^, Sigma-Aldrich, catalog no. H9268) for 24 h. The cells were then infected again for 24 h. Resistant clones were cultured, and the efficiency of knockdown or overexpression was confirmed by quantitative reverse transcriptase-polymerase chain reaction (qRT-PCR) or western blots. In rescue experiments, DMSCs with knockdown were transduced with retroviral constructs containing the Flag- gene of interest. The target sequences for *shRNA* were as follows: *sh1KDM6B*: 5′- GCAGTCGGAAACCGTTCTT −3′, *sh3KDM6B*: 5′- GTGGGAACTGAAATGGTAT -3′. Flag-*KDM6B* full-length cDNA was cloned into a retroviral construct by PCR.

### Alkaline phosphatase staining and ALP activity assay

For ALP staining, cells were fixed with 70% ethanol and incubated with a solution of 0.25% naphthol AS-BI phosphate (Sigma-Aldrich, catalog no. N6125) and 0.75% Fast Blue BB (Sigma-Aldrich, catalog no. D9805), which was dissolved in 0.1 mol·L^-1^ Tris buffer (pH 9.6) after 5–7 days of induction. ALP activity assays were performed using alkaline buffer solution (Sigma-Aldrich, catalog no. A9226) with alkaline phosphate yellow liquid substrate (Sigma-Aldrich, catalog no. P7998) for 30 min at 37 °C, and the absorbance of the reactions was read at 405 nm and then normalized based on protein concentrations.

### Alizarin red S staining

For detection of the mineralization potential, cells were induced for 2–3 weeks, fixed with 70% ethanol in 1 h and stained with 1% Alizarin Red S (Sigma-Aldrich, catalog no. A5533). For quantification of calcium mineral deposition, Alizarin Red S was destained using 10% cetylpyridinium chloride in 10 mmol·L^-1^ sodium phosphate for 30 min at room temperature. The concentration was determined by absorbance measurement at 562 nm on a multiplate reader and calculated by a standard calcium curve in the same solution. The final calcium level was normalized to the total protein concentrations in each group prepared from a duplicate plate.

### qRT-PCR

Total RNA was isolated from DMSCs using TRIzol reagents (Thermo Fisher Scientific, catalog no. 15596018). RNA was synthesized from cDNA with random hexamers and reverse transcriptase according to the manufacturer’s protocol (New England Biolabs, catalog no. E6300). qRT-PCRs were performed using the SYBR Green PCR kit (New England Biolabs, catalog no. E3005) and the CFX qRT-PCR Detection System. Primer pairs for qRT-PCR were as follows: *ALP*: forward, 5′- GACCTCCTCGGAAGACACTC -3′; reverse, 5′- TGAAGGGCTTCTTGTCTGTG -3′. *OSX*: forward, 5′- GGAAGAAGCCCATCCACA -3′; reverse, 5′- AAGCCTTGCCATACACCTTG -3′. *DLX5*: forward, 5′- CTACAACCGCGTCCCAAG -3′; reverse, 5′- GCCATTCACCATTCTCACCT -3′. *KDM6B*: forward, 5′- CCTCGAAATCCCATCACAGT -3′; reverse, 5′- GTGCCTGTCAGATCCCAGTT -3′. *OCN*: forward, 5′- TGAGAGCCCTCACACTCCTC -3′; reverse: 5′- TCCTGCTTGGACACAAAGG -3′. *HOXC6*: forward, 5′- GAATTCCTACTTCACTAACCC-3′; reverse, 5′-TCATAGGCGGTGGAATTGAG-3′. The primers for epigenetic gene profiling are provided in Supplementary Table 1.

### Chromatin immunoprecipitation-qPCR (ChIP-qPCR) assays

For each ChIP reaction, 2 × 10^6^ cells were used. Cells were incubated with a dimethyl 3,3′ dithiobispropionimidate-HCl (DTBP) (Pierce Biotechnology, catalog no. 20665) solution (5 mmol·L^-1^) for 10 min at room temperature in a dark area and treated for 15 min at 37 °C in 1% formaldehyde. Total cell lysates were sonicated to generate ~200–500 bp DNA fragments. All resulting precipitated samples were quantified using qPCR. Data are expressed as a percentage of input DNA. Antibodies used for ChIP assays were purchased from the following commercial sources: rabbit polyclonal anti-KDM6B (Abcam, catalog no. ab85392), rabbit monoclonal anti-H3K27me3 (Millipore Sigma, catalog no. 07–449), and rabbit monoclonal anti-ERα (Abcam, catalog no. ab32063). Primer pairs were *HOXC6*: forward, 5′- GGTTGTGCCACAGTCTAGAAG -3′; reverse, 5′- TTTCACAATGGGCAGCGGG -3′. *BMP2*: forward, 5′- TCCTCTGAGCTGCTAATCGC -3′; reverse, 5′- TGTGGCAGACTGAAGGATGG -3′. *KDM6B*: forward, 5′- CCCAGTAAGGTCAGATGTGGTC -3′; reverse, 5′- TGGGAGAAGGACTAGGGATG -3′.

### Protein extraction and western blot

Cells were treated with E2 (10 nmol·L^-1^) in OIM. After 24 and 48 h, cells were collected and washed once using PBS. RIPA buffer (150 mmol·L^-1^ NaCl, 50 mmol·L^-1^ Tris-Cl, 1% NP-40, 0.5% DOC, 0.1% SDS, 1:100 PMSF and 1:100 PImix) was used to lyse cells for 30 min at 4 °C. The supernatant was collected as total protein after centrifugation at 12 000 r·min^-1^ for 20 min. Equal amounts of protein were separated by SDS-PAGE and detected for target proteins. Antibodies against ERα (1/1 000, Abcam, Cambridge, MA, USA, ab32063) and β-actin (1/2 000, Sigma, St. Louis, MO, USA, A1978) were utilized.

### Immunofluorescence staining

Cells were grown on 10 mm glass coverslips. After OIM with or without E2 (10 nmol·L^-1^) treatment for 4 h, the cells were fixed in fresh 4% paraformaldehyde (PFA). The coverslips were washed with PBS for 5 min twice. The cells were then blocked in 1% BSA for 1 h at room temperature, followed by incubation with primary antibody overnight in a 4 °C refrigerator (ERα, 1/200). On the following day, coverslips were washed with PBS for 3 min twice. Then, secondary antibodies were applied to the cells for 1 h at room temperature. After PBS washing, the nucleus was stained with DAPI. The cells were visualized under fluorescence or confocal microscopy.

### Scaffold preparation

Apatite-coated poly(lactic-coglycolic acid) (Ap-PLGA) scaffolds were prepared through solvent casting and particulate leaching processes following an established protocol as previously described.^65,66^ Briefly, PLGA/CHCl_3_ solution was mixed with sucrose (200–300 µm in diameter) to achieve a porosity of 92% (v/v), and the slurry was compressed into Teflon molds. After being lyophilized overnight, the scaffolds were immersed in Milli-Q water to leach out sucrose. The scaffolds were then sterilized in 70% EtOH for 30 min and rinsed with sterile water. Last, the scaffold sheets were shaped into round discs (3 mm in diameter and 0.5 mm in height) with a biopsy punch. The PLGA scaffolds were further coated with apatite layers by incubating scaffolds in simulated body fluid (SBF). The scaffold discs were subjected to glow discharge argon plasma etching (Harrick Scientific). The etched scaffolds were incubated in SBF1 composed of CaCl_2_, MgCl_2_•6H_2_O, NaHCO_3_, K_2_HPO_4_•3H_2_O, Na_2_SO_4_, KCl and NaCl for 24 h and then further incubated in SBF2 composed of CaCl_2_, K_2_HPO_4_•3H_2_O, KCl and NaCl for another 24 h at 37 °C. The Ap-PLGA scaffolds were loaded with phosphate-buffered saline (PBS) or E2 (1, 5, 25 μmol·L^-1^) and lyophilized for further usage.

### Animals and surgical implantation

The animal protocol for this study was approved by the Animal Research Committee at the University of California, Los Angeles. Eight-week-old male SCID mice (Charles River Laboratories) were randomly divided into four groups. In each mouse, a 3-mm-diameter calvarial critical-sized defect was created on the parietal bone with a trephine drill as previously described.^67^ After gentle removal of the circular bone plug, DMSCs (1 × 10^6^ cells) seeded with or without the E2 scaffold were placed on the defect. After surgery, all animals were allowed to fully recover. All mice were euthanized 8 weeks after implantation.

### Micro-CT analysis

The calvarium was dissected and fixed with 10% v/v formalin for 48 h. High-resolution microcomputed tomography (SkyScan 1275; Bruker) was used to scan samples at a resolution of 10 μm with a 60-kV and 166-μA X-ray source and a 1-mm aluminum filter. For the analysis, NRecon software (Bruker) with image correction steps was used to reconstruct three-dimensional image datasets from 2D X-ray images, DataViewer software (Bruker) was used to orient with images on a 3D plane, and CTAn software (Bruker) was used to conduct three-dimensional volumetric analysis. The region of interest was outlined with a circular shape on consecutive transaxial sections to create a uniform volume of interest. BV/TV values were quantified.

### Histological analysis

Samples were fixed in 4% PFA for 24 h and decalcified in 10% ethylenediaminetetraacetic acid (0.1 mol·L^-1^, pH 7.1) solution for 2 weeks. After decalcification, the parietal bone was removed from the cranium and embedded in paraffin. Tissue samples were sectioned into 5-μm sections and stained with hematoxylin and eosin at the University of California, Los Angeles, Tissue Procurement Core Lab. Slides were submerged in 0.1% trypsin diluted in 1X PBS for 10–20 min at 37 °C for antigen retrieval and then incubated with anti-KDM6B antibody (1:100, Abcam, catalog no. ab85392) as a primary antibody. Tissue sections were visualized using 3-amino-9-ethylcarbazole (AEC; Vector Laboratories), followed by counterstaining with hematoxylin. Sections were sealed with Faramount aqueous mounting solution (Dako) as previously described.^68^

An Olympus IX73 microscope and cellSens software version 1.6 (Olympus Corporation) were used to visualize and quantify the bone volume of calvarial defects in IHC. Three random fields of view per section sample were randomly taken for each specimen, and the average values were used as the data point for each specimen.

### Statistical analysis

Data are expressed as the mean ± SEM. Differences in measured variables between two groups were assessed by two-tailed Student’s *t*-tests. One-way ANOVA with Tukey’s post hoc test was applied when there were more than two groups. Two-way ANOVA with a Bonferroni post hoc test was applied when there were multiple groups across different points. When a normal data distribution could not be assured, the Wilcoxon/Mann–Whitney test was applied. Using GraphPad Prism 5, the results were considered statistically significant at *P* < 0.05.

## Supplementary information

Supplementary Information

## Data Availability

The data that support the findings of this study are available from the corresponding author upon reasonable request.
