# Supplementary material for: The ERα/KDM6B regulatory axis modulates osteogenic differentiation in human mesenchymal stem cells
Source: Bone Res. 2022 Jan 7;10:3. doi: 10.1038/s41413-021-00171-z (PMC8738748; doi:10.1038/s41413-021-00171-z)
Supplement: Supplementary file 1 — Supplementary Information [file 41413_2021_171_MOESM1_ESM.pdf]

SUPPLEMENTARY INFORMATION FOR

ERα/KDM6B Regulatory Axis Modulates Osteogenic Differentiation in Human Mesenchymal Stem Cells

Zhenqing Liu, Hye-Lim Lee, Jin Sook Suh, Peng Deng, Chang-Ryul Lee, Olga Bezouglaia, Mojan Mirnia, Vivian Chen, Michael Zhou, Zhong-Kai Cui, Reuben H. Kim, Min Lee, Tara Aghaloo, Christine Hong*, Cun-Yu Wang*

* Correspondences:
Christine Hong (yeumin.hong@ucsf.edu) and Cun-Yu Wang (cwang@dentistry.ucla.edu)

This PDF includes

Supplementary Methods

Supplementary Figures with Legends Fig. S1 and 2

Supplementary Methods

Multi-lineage differentiation potential of DMSCs

DMSCs at passage 5-7 (6×10^4 cells) were seeded into 24-well plates. For osteogenic differentiation, DMSCs were differentiated in α-MEM with 10% FBS containing 100 μM ascorbic acid, 2 mM β-glycerophosphate, and 10 nM dexamethasone. for 14 days. Differentiated cells were fixed in 10% Neutral buffered formalin for 10 min and stained with 2% Alizarin Red solution for 20 min at room temperature to visualize calcium deposits. For adipogenic differentiation, DMSCs were differentiated in α-MEM with 10% FBS containing 0.5 mM isobutylmethylxanthine, 0.5 μM hydrocortisone, and 60 μM indomethacin (Sigma-Aldrich) for 21 days. Differentiated cells were fixed in 10% Neutral buffered formalin for 10 min and stained with 0.5% Oil Red O solution for 15 min at room temperature to detect lipid vacuoles. For chondrogenic differentiation, DMSCs were differentiated in α-MEM with 10% FBS containing 100 mM sodium pyruvate, 40 μg/ml proline, 100 nM dexamethasone, 200 μM ascorbic acid (all from Sigma-Aldrich), and 10 ng/mL TGF-β (R&D systems) for 21 days. Differentiated cells were fixed in 10% Neutral buffered formalin for 10 min and stained with 1% Alcian Blue 8GX solution (Sigma-Aldrich) for 30 min at room temperature to observe the proteoglycan content. The differentiation assay was performed two times with triplicated samples.

Supplementary Fig. 1

Supplementary Fig. 1

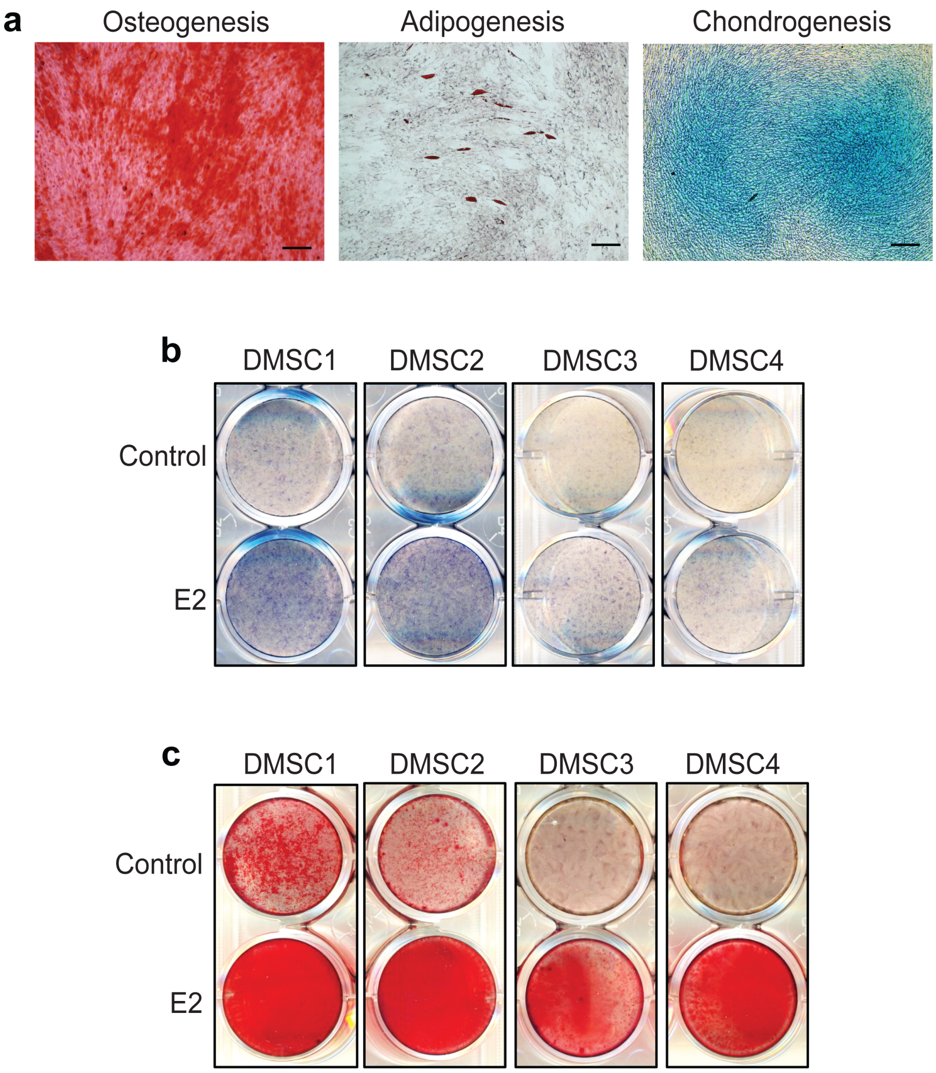

Supplementary Figure 1. E2 increases osteogenic differentiation on DMSCs. **a** Multipotency assessment of DMSCs. Left: Alizarin Red S staining after 14 days treatment of osteogenic media; Middle: Oil red O staining after 21 days treatment of adipogenic media; Right: Alcian blue staining after 21 days treatment of chondrogenic media. Scale bar: 100μm. **b** Alkaline phosphatase staining and quantification after 5 days treatment of osteogenic media with and without E2 on DMSCs from four different donors. **c** Alizarin Red S staining and quantification after 14 days treatment of osteogenic media with and without E2 on DMSCs from four different donors.

Supplementary Fig. 2

Supplementary Fig. 2

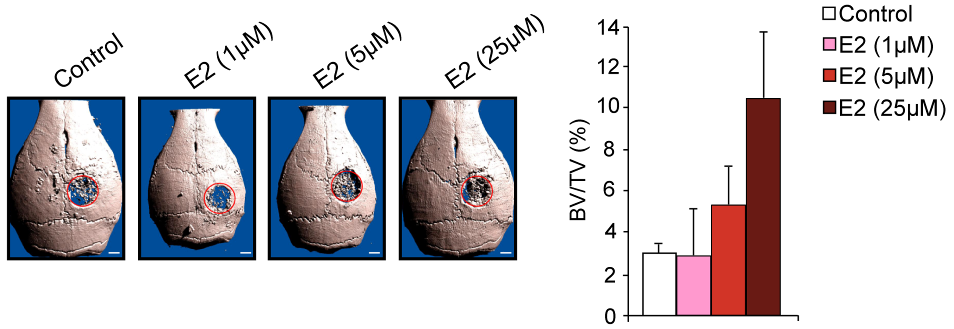

Supplementary Figure 2. E2 increased DMSC bone formation *in vivo*. Micro-CT images and bone volume quantification (BV/TV) of calvarial bone defects with scaffolds containing DMSCs with different concentration of E2 (1μM, 5μM, 25μM) and control. Data are presented as means ± SEMs (*n* = 3). Scale bars, 1mm.

Supplementary Table 1

The primers for epigenetic regulator genes are as follows:

Genes	Forward	Reverse
KDM2A	CAAGGAGAGTGTGGTGTTTGCC	ACCTCTCCACAGAGGGAACATG
KDM2B	CATGGAGTGCTCCATCTGCAATG	ACTTCGGACACTCCCAGCAGTT
KDM3A	GCCAACATTGGAGACCACTTCTG	CTCGAACACCTTTGACAGCTCG
KDM3B	GCTCGTAATGTCTGAGAAGGAGG	CACATTTGCGACAAACCCAGTGG
KDM3C	TCCTGTCAGACCTTCCAGTGCA	GTGGATGCAACAGACCGTAATGG
KDM4A	TGCGGCAAGTTGAGGATGGTCT	GCTGCTTGTTCTTCCTCCTCATC
KDM4B	GCCGAGAGGAAGTTCAACGCAG	TGCCTCCTTCTCAGTCTGTAGG
KDM4C	CCGATGACTCTTGTGAAGCAGC	GACTTCGTCTGCCAAAGGTGGA
KDM4D	CCTGAACGCTATGACCTGTGGA	TCTCCTGGGTAACTGGACTTCC
KDM5A	GCTAAGGTCTGCCTACAGGCAA	CCACTTTAGCGGTCCATTCTCG
KDM5B	AGCCAGAGACTGGCTTCAGGAT	AGCCTGAACCTCAGCTACTAGG
KDM5C	ACTGCTGACCATTGCTGAACGC	CCTCCTTGAGAGCCTGGATGTT
KDM5D	GGCTGAGTCTTTTGACACCTGG	CAGGCAGTTCTTCAGTCGCTGA
KDM6A	AGCGCAGAGGAGCCGTGGAAAA	GTCGTTCACCATTAGGACCTGC
KDM6B	CCTCGAAATCCCATCACAGT	GTGCCTGTCAGATCCCAGTT
KDM6C	GAGAAAACAGCTATCTAACTCCGC	CAGTGCTAGTTGCCTGGTGATAC
KDM7A	CGGTGGAACTTCAGTCTGGTAC	CTCCAAAGAACACCTCACTCTGG
KDM7B	GGACACATACAGTCATCAGGCAC	GGCTCTCATTTCCATCAAGGTCC
KDM7C	TGCGTGAAGGACAGTTACACCG	CATCTCGCTGTGGTTAGAGGCA
HR	TCTGCGAACTGCTGGCTTCTAC	GTGCGATAATGCTGTCCAGGATG
JARID2	GGACAAAGGCGTCCTCAATGAC	GCAGGCTCCTTGCTGAAACACA
JMJD4	ACTTCTCGTCCGACTGGCTGAA	AGCTGAAGGAGCGGAAGATGTC
JMJD5	CACAGATGAGGAATGGTCCCAG	GCTGATGTCCTGCTTCAACTCC
JMJD6	CCAACTTCCCTGTGGTATGGCA	TCCTGAAGGTCAACCGAGTCTG
JMJD7	GGAGTCCTCTATGTGCAGAAGC	CAGCCAGAAGTTCACAGCATCG
